# High night temperature during the effective grain-filling stage affects maize yield and quality

**DOI:** 10.3389/fpls.2026.1826388

**Published:** 2026-07-03

**Authors:** Shumei Wang, Teng Li, Shuo Liu, Sai Zhang, Peng Sui, Xuepeng Zhang

**Affiliations:** 1 Crop Research Institute, Shandong Academy of Agricultural Sciences, Jinan, China; 2 College of Agronomy, China Agricultural University, Beijing, China

**Keywords:** grain filling, grain yield and quality, high night temperature, photosynthesis, *Zea mays* L.

## Abstract

Global warming has led to a disproportionate increase in night temperature, imposing additional constraints on crop growth and yield. However, how maize (*Zea mays* L.) responds to high night temperature (HNT) during the effective grain-filling stage remains unclear. This study aimed to determine how HNT affects maize yield, grain quality, photosynthesis–respiration coordination, and starch metabolism. A temperature-controlled experiment with heat-sensitive hybrid (ZY 309) and tolerant hybrid (JH 5) was carried out. Night temperature treatments of 24 °C (control), 26 °C, 28 °C and 30 °C were applied for 18 consecutive days encompassing the effective grain-filling stage. The grain yield of ZY 309 decreased significantly by 9.27% and 13.64% at 28 °C and 30 °C, and that of JH 5 decreased significantly by 11.19% at 30 °C. At 30 °C, grain starch content decreased by 2.27% in ZY 309 and 2.70% in JH 5, lipid content decreased by 11.37% and 10.61%, respectively, whereas protein content increased by 11.46% and 9.92%, and coarse fiber content increased by 10.56% and 5.30%. Although leaf photosynthesis of ZY 309 remained 27.19% and 39.00% higher than the control at 28 °C and 30 °C, this increase did not prevent yield loss. Night respiration increased with temperature and treatment duration, with a stronger response in ZY 309. The expression levels of *AM-AGPase SS* and *AM-AGPase LS* were more significantly reduced in ZY 309 than JH 5. These results indicate that HNT during effective grain filling reduces maize yield and grain quality, mainly by increasing respiratory carbon cost and weakening starch biosynthesis, with ZY 309 showing greater sensitivity than JH 5.

## 1 Introduction

By the end of the 21st century, it is anticipated that the global average temperature will increase by 0.3 °C to 4.8 °C, leading to irreversible impacts on crop production (IPCC, 2021). Due to the asymmetry increase of temperature, night temperatures increase at a rate of 1.4 times that of daytime temperatures (Alexander et al., 2006; Davy et al., 2017; Sillmann et al., 2013). At the same time, with the increase of cloud cover at night, radiant heat loss is greatly reduced, resulting in a longer duration of temperature rise at night (Alward et al., 1999). Consequently, the increase in night temperatures can also cause serious harm to crop production.

A series of existing studies have demonstrated that night warming is detrimental to crops such as rice (Bahuguna et al., 2017; Welch et al., 2010), wheat (McAusland et al., 2021), barley (García et al., 2015), soybean (Yang et al., 2023) and maize (Hein et al., 2024; Kettler et al., 2022; Wang et al., 2020). Research indicates that an increase in night temperature from 20 °C to 30 °C during the wheat seedling stage results in a dramatic 60% reduction in leaf area (Schoppach and Sadok, 2013). Moreover, high night temperatures also affect daytime photosynthetic capacity by affecting night carbohydrate accumulation and chlorophyll degradation (Mohammed and Tarpley, 2009; Turnbull et al., 2002; Vågen et al., 2003). Not only do high night temperatures affect crop growth and development, but they also impair yield and quality. Retrospective analysis has shown that in the dry season, a 1 °C rise in the minimum night temperature correlates with a 10% reduction in both rice biomass production and grain yield (Peng et al., 2004). The penalty is particularly evident in wheat: the yield of 11 wheat genotypes decreased by 20.3% on average when night temperature increased by 3.2 °C during the grain-filling stage (Hein et al., 2019). As for quality, high night temperature increased the chalkiness of rice and decreased the amylose content in grain (Lanning et al., 2011; Shi et al., 2013). Wheat experienced night temperature greater than 23 °C during the flowering and filling stage, which resulted in increased activity of starch enzyme in grain (Impa et al., 2020).

Although maize is considered a thermophilic crop, its response to high night temperature remains insufficiently understood, particularly during the effective grain-filling stage. Previous studies have mainly focused on the flowering stage, where high night temperature was shown to reduce pollen viability, shorten pollen shedding duration, and decrease kernel number (Cantarero et al., 1999; Wang et al., 2020). However, some studies have also examined the post-flowering stage, showing that a moderate increase in night temperature (2.3 °C) reduced radiation use efficiency, while a larger increase (4.3 °C) further decreased leaf photosynthesis (Kettler et al., 2022). More recently, differential effects of daytime and nighttime high temperature on waxy maize starch structure and physicochemical properties have been reported, indicating that nighttime heat can directly affect starch accumulation and amylopectin structure during grain development (Li et al., 2025). In contrast, much less is known about the effective grain-filling stage, during which most of the final kernel dry matter is accumulated. This stage is critical not only for kernel weight determination, but also for grain compositional quality (Daynard et al., 1971). Moreover, previous studies on post-flowering night warming have mostly emphasized yield or general carbon balance responses, whereas the effects of high night temperature on leaf photosynthesis-respiration coordination, grain quality formation, and starch metabolism regulation during the effective grain-filling stage remain unclear (Li et al., 2022). Taken together, a key unresolved issue is how elevated night temperature during the effective grain-filling stage alters maize yield formation and grain quality.

Therefore, the present study focused on maize during the effective grain-filling stage and aimed to determine how elevated night temperature influences yield formation and grain quality, how it affects the coordination between leaf photosynthesis and respiration, and whether starch metabolism differs between heat-sensitive and heat-tolerant hybrids. We hypothesized that elevated night temperature during the effective grain-filling stage would reduce kernel weight and grain yield in a hybrid-dependent manner, primarily through increased respiratory carbon cost and impaired starch biosynthesis, ultimately leading to changes in grain-quality composition.

## 2 Materials and methods

### 2.1 Experiment setup

The pot experiments were performed at the Wuqiao Experimental Station of China Agricultural University (37°41′ N, 116°37′ E), Hebei Province, China. The site is located in the North China Plain and is characterized by a temperate continental monsoon climate. During the experimental period before the temperature treatments, the mean air temperature and total precipitation were approximately 26 °C and 180 mm. Two hybrids were utilized, Zhuyu 309 (ZY 309), previously reported as a heat-sensitive hybrid (Wang et al., 2020), and Jinhai 5 (JH 5), a high-yielding hybrid that showed relatively higher heat tolerance in previous field experiments (Yan et al., 2017). Three seeds were sown in each pot (32 cm in diameter and 34 cm in height) filled with a mixture of clay soil and vermiculite at a ratio of 3:1. At the sowing stage, each pot received 4 g of basal fertilizer (N/P_2_O_5_/K_2_O at 23/15/18%), and at the six-leaf stage, 4 g of urea (N at 46%) was applied per pot. At the three-leaf stage, the seedlings were thinned out, leaving only one plant per pot. For each hybrid, 40 pots were prepared and evenly assigned to the four night-temperature treatments, resulting in 10 pots per hybrid per treatment. Each pot contained one plant. Within each temperature-controlled greenhouse, 20 pots were arranged, including 10 pots of ZY 309 and 10 pots of JH 5. Six plants per hybrid per treatment were used for repeated non-destructive physiological measurements during the treatment period and for final yield and grain-quality determination at maturity. Three plants per hybrid per treatment were sampled at 12 DAHNT for grain hormone content, enzyme activity, and gene expression analyses. Before temperature control treatment, pots were uniformly managed for outdoor growth. During the outdoor growth period and the treatment, soil water availability was maintained under non-water-limited conditions. Pots were weighed daily, and irrigation was adjusted to maintain soil water content at approximately 75–80% of field capacity. Weeds were removed manually when present throughout the experiment. Plants were inspected regularly for visible symptoms of insect pests and diseases. No severe pest or disease incidence was observed during the treatment period, and no chemical pesticide was applied during the treatment.

### 2.2 Temperature treatments

**Figure 1:**
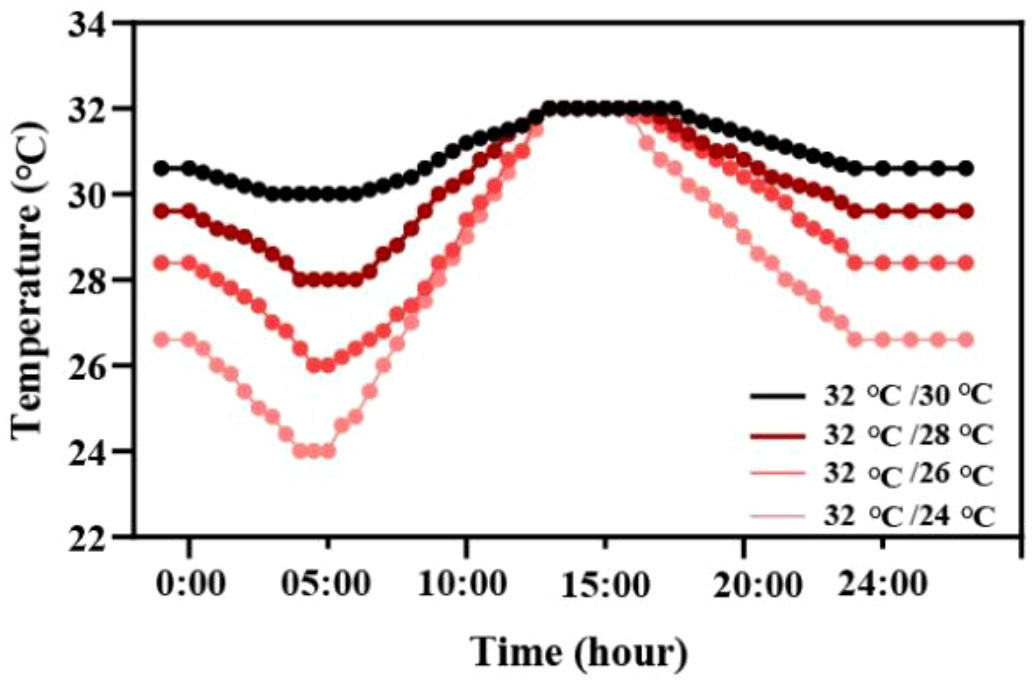
Diurnal temperature dynamics during a representative day under the 24 °C (control), 26 °C, 28 °C, and 30 °C night temperature treatments.

At 15 days after silking, the potted plants were transferred to four intelligent temperature-controlled glass greenhouses for continuous temperature control, with solar transmittance of 85–90% in the greenhouses. Within each compartment, pots of the two hybrids were arranged randomly, and their positions were adjusted daily during the treatment to reduce positional effects caused by uneven illumination. All compartments had the same structure, similar solar transmittance, pot arrangement, substrate, fertilization, irrigation, and crop management practices. The day and night temperatures of the greenhouses were set to simulate the trend of atmospheric temperature changes. The daily maximum temperature of the four treatments was set at 32 °C, and the daily minimum temperatures were set at 24 °C (control), 26 °C, 28 °C and 30 °C, respectively. The daily maximum temperature was set at around 15:00, and the daily minimum temperature was set at 05:00–06:00 (**Figure 1**).

### 2.3 Sampling and measurements

Maize plants entered the silking stage at 65 days after sowing. After the maize had passed the flowering stage and lag phase (about 15 days after silking), the plants were transferred to the greenhouse for night heating. The heating treatment lasted for 18 consecutive days, and samples were collected at 6, 12, and 18 days after high night temperature treatment (DAHNT) (**Supplementary Figure 1**). Plants used for yield determination were harvested 6 days after the end of the heating treatment, when they reached physiological maturity.

#### 2.3.1 Grain yield and quality

Plants used for grain yield determination reached physiological maturity 6 days after the end of the 18-day heating treatment. Six plants per treatment were harvested to assess grain yield, kernel weight and kernel number per ear. Ears were harvested from each plant and manually threshed. All kernels from each ear were manually counted to determine kernel number per ear. The kernels were then dried at 80 °C to constant weight, and grain yield was expressed on a dry weight basis as g plant^-^¹. Kernel weight was determined for each biological replicate using the 100-kernel weight method and expressed as g grain^-^¹. Grain protein, lipid, and coarse fiber content were quantified using the DA7250 NIR (near-infrared) analyzer instrument (Perten Instruments, Hägersten, Sweden). The amylose contents of grains were calculated using the method described by Kasemsuwan et al. (1995). Following the procedure described by Jane and Chen (1992), isoamylase was used to debranch amylopectin.

#### 2.3.2 Leaf photosynthesis, chlorophyll fluorescence, and night respiration rate

During the treatment period, at 6, 12, and 18 DAHNT, net photosynthetic rate of the ear leaf was measured on six randomly selected plants per treatment for each hybrid between 09:00 and 12:00 using a LI-6400 portable photosynthesis system (LI-COR, Lincoln, NE, USA). Night respiration rate was measured on the same leaves between 22:00 and 24:00 on the same day. During photosynthetic measurements, the leaf chamber conditions were set at 32 °C, 400 μmol mol^-^¹ CO_2_, 1000 μmol m^-^² s^-^¹ photosynthetically active radiation, and a flow rate of 500 μmol s^-^¹. Net photosynthetic rate (Pn), stomatal conductance (Gs), intercellular CO_2_ concentration (Ci), and transpiration rate (Tr) were recorded simultaneously. For respiration measurements, the CO_2_ concentration was maintained at 400 μmol mol^-^¹. Chlorophyll fluorescence was assessed using a portable pulse fluorometer MINI-PAM II (Heinz Walz, Germany, 2017), with six replicates per treatment. Before measurement, the ear leaves were pre-darkened for 30 minutes at 6, 12, and 18 DAHNT. The minimum fluorescence (F0), the induction of maximum fluorescence (Fm), the steady-state fluorescence yield (Fs), the light-adapted maximum fluorescence (Fm′) and the light-adapted minimum fluorescence (F0′) were measured according to Zhang et al. (2020).

Variable fluorescence was calculated as Fv = Fm − F0.

The maximum quantum efficiency of PSII was calculated as Fv/Fm = (Fm − F0)/Fm.

Photochemical quenching was calculated as qP = (Fm′ − Fs)/(Fm′ − F0′).

Non-photochemical quenching was calculated as NPQ = (Fm − Fm′)/Fm′.

#### 2.3.3 Grain hormone content and enzyme activity

For grain hormone content, enzyme activity, and gene expression analyses, samples were collected only from the 24 °C and 30 °C treatments at 12 DAHNT. These treatments represented the control and the highest night temperature condition, respectively, and were selected to compare physiological and molecular responses under normal and severe HNT conditions. Grains were sampled from the middle region of three ears per treatment at 12 DAHNT for the determination of hormone content and amylase activity. Grain samples (0.5 g) were used to measure the contents of cytokinin (CTK), indole-3-acetic acid (IAA), gibberellic acid (GA), and abscisic acid (ABA) according to Zhang et al. (2020), performed at the Phytohormones Research Institute, China Agricultural University, using an ELISA reader (model EL310, Bio-TEK, Winooski, VT) according to the manufacturer’s instructions. In addition, α-amylase and β-amylase activities were measured using a commercially available amylase activity test kit.

#### 2.3.4 Starch metabolism enzymes expression profile

For gene expression analysis, grains were collected from the middle region of three ears per treatment at 12 DAHNT under the 24 °C and 30 °C night temperature treatments. Total RNA was extracted from the sampled grains and reverse-transcribed into cDNA using the PrimeScript RT Reagent Kit (Takara, China). Quantitative real-time PCR (qRT-PCR) was performed on a QuantStudio 6 Flex System (Thermo Fisher Scientific, China) using SYBR Green dye (Takara, China). The gene-specific PCR primers (forward and reverse) used for expression analysis are detailed in **Supplementary Table 2**. Actin was used as the reference gene for normalization of qRT-PCR data. The relative quantitative method was employed to assess the differences in target mRNA expression. The 2^-ΔΔCT^ method was applied to quantify the relative expression of the target gene mRNA, as shown in **Supplementary Table 3**. For hormone content and gene expression data, the same assumption checks were performed before ANOVA, and transformed values were used when required.

### 2.4 Statistical analysis

The statistical analysis was performed using SPSS 26.0 (SPSS Inc., Chicago, IL, USA). To evaluate the significance of treatment effects, a two-way ANOVA was conducted using hybrid (H) and night temperature treatment (T) as fixed factors. Mean among temperature treatments within each hybrid were performed using Fisher’s least significant difference (LSD) test at P < 0.05. The F-values, degrees of freedom, and P-values of the ANOVA are provided in **Supplementary Table 5**.

## 3 Results

### 3.1 Grain yield and quality of maize

**Figure 2:**
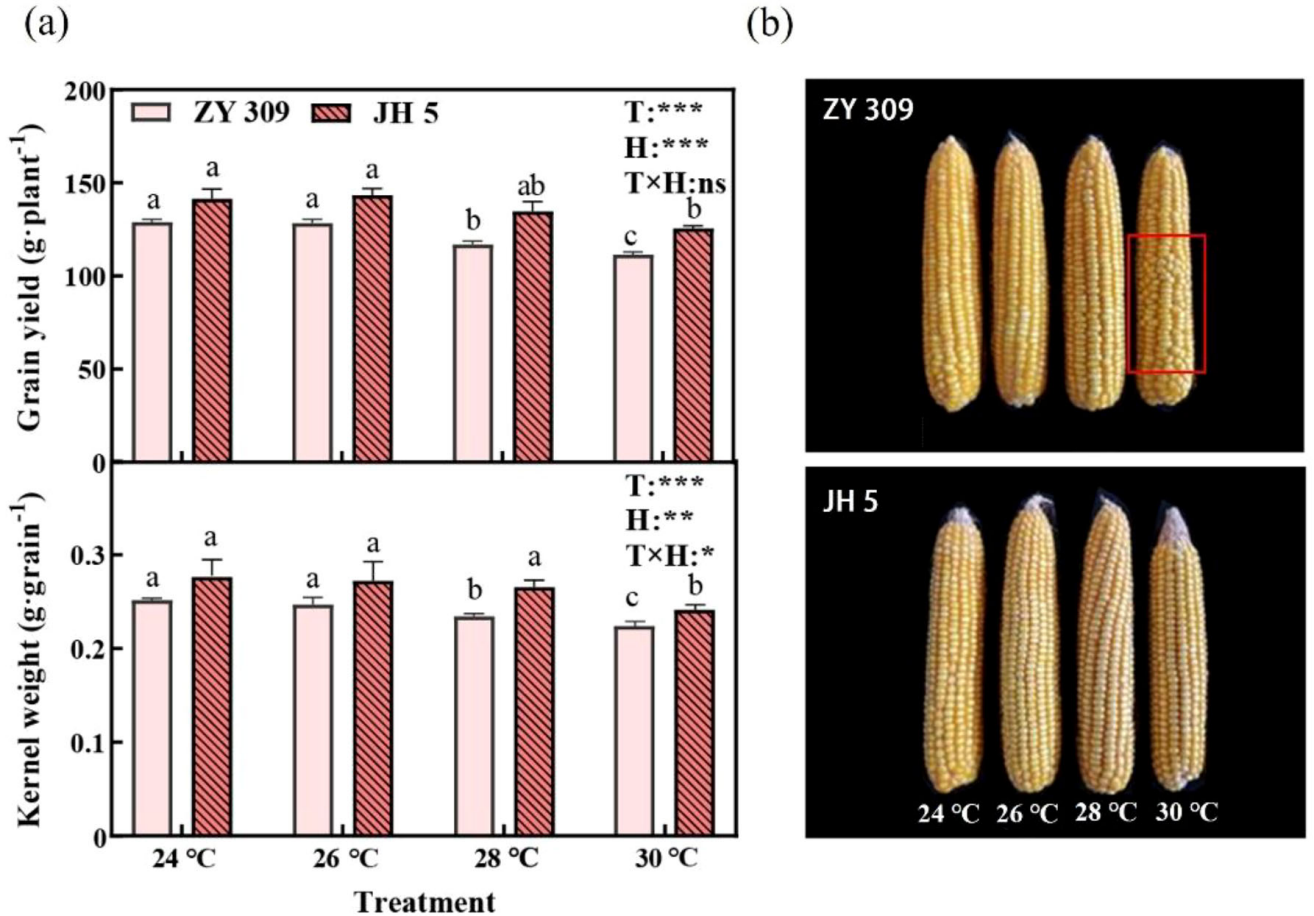
**(a)** Grain yield and kernel weight of ZY 309 and JH 5 under different night temperature treatments. **(b)** Representative ears of ZY 309 and JH 5 under different night temperature treatments. Data are presented as means ± standard deviation (SD) of six biological replicates. Different lowercase letters indicate significant differences among treatments within the same hybrid at *P* < 0.05. T represents the temperature factor, H represents the hybrid factor, and T × H indicates the interaction effect. ***, **, and * indicate significance at *P* < 0.001, *P* < 0.01, and *P* < 0.05, respectively; ns indicates no significant difference.

With the increase in night temperature, the grain yield of both hybrids showed a gradual decreasing trend (**Figure 2**). However, compared with the control (24 °C), the 26 °C treatment did not significantly affect grain yield in either hybrid. Grain yield of ZY 309 decreased significantly by 9.27% and 13.64% at 28 °C and 30 °C, respectively, whereas grain yield of JH 5 decreased significantly by 11.19% only at 30 °C. Since the night-heating treatments were imposed after silking, kernel set was not expected to be affected. Consistently, kernel number per ear did not differ significantly among temperature treatments within the same hybrid (**Supplementary Figure 2**). Therefore, the reduction in grain yield was mainly associated with the decrease in kernel weight. In line with the grain yield response, kernel weight was not significantly affected by the 26 °C treatment in either hybrid. In ZY 309, kernel weight decreased significantly by 6.84% and 10.94% at 28 °C and 30 °C, respectively, compared with 24 °C. In JH 5, kernel weight decreased significantly only at 30 °C, reaching a minimum value of 0.24 g grain^-^¹. These results indicate that ZY 309 was more sensitive to elevated night temperature than JH 5 during the effective grain-filling stage. In addition, phenotypic observation of ears showed abnormal grain development in ZY 309 under the 30 °C treatment.

**Figure 3:**
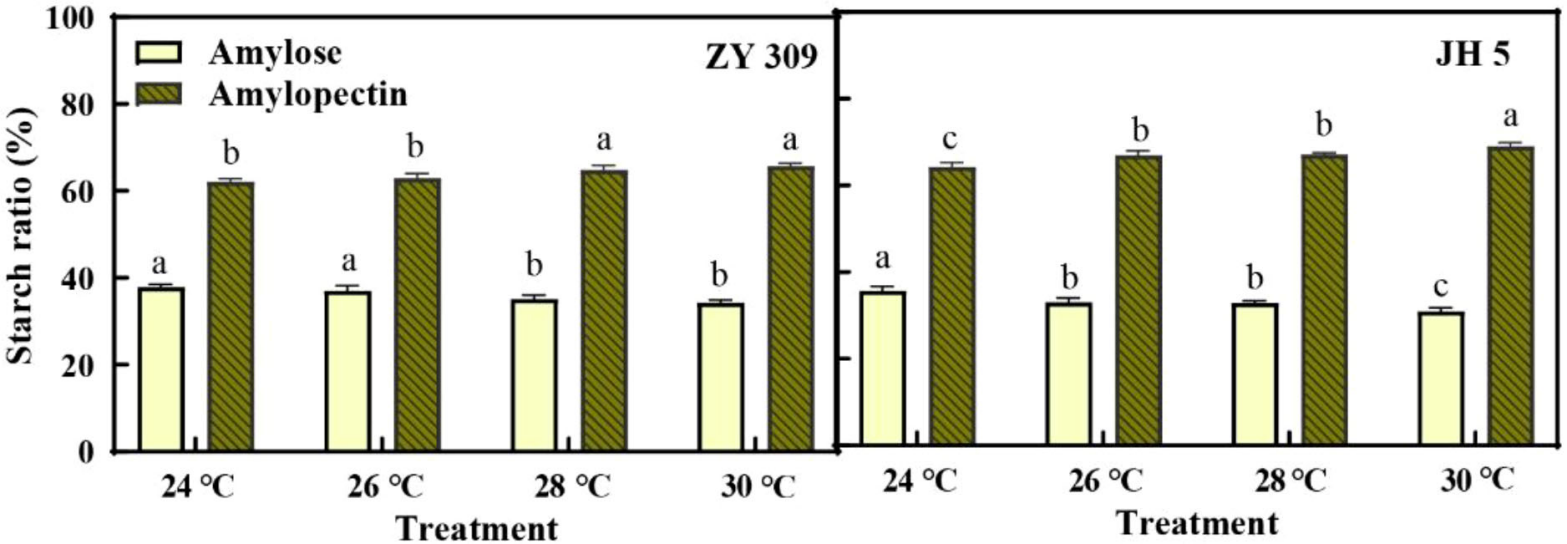
Amylose and amylopectin ratio of ZY 309 and JH 5 under different night temperature treatments. Data are presented as means ± standard deviation (SD) of six biological replicates. Different lowercase letters indicate significant differences among treatments within the same hybrid at *P* < 0.05.

The grain starch proportion of ZY 309 at 30 °C was significantly reduced by 2.27%, and that of JH 5 at 30 °C was significantly lower than that at 24 °C (2.70%). The proportion of amylopectin content in the grains of both hybrids increased significantly with the increase of night temperature (**Figure 3**). The high temperature at 28 °C and 30 °C significantly increased the amylopectin content in grains of ZY 309 by 4.41% and 5.73%, respectively, and the high temperature at 30 °C significantly increased the amylopectin content in JH 5 grains by 7.36%.

With the increase in night temperature, the protein content of ZY 309 and JH 5 increased by 11.46% and 9.92% respectively to the maximum under 30 °C (**Table 1**). Meanwhile, the protein content of ZY 309 was higher than that of JH 5. The percentage of grain lipid content in ZY 309 decreased by 3.32% and 11.37% at 28 °C and 30 °C, respectively, while that in JH 5 decreased by 10.61% at 30 °C. The coarse fiber proportion of ZY 309 at 28 °C and 30 °C was significantly higher than that of the control (11.22% and 10.56%), and that of JH 5 at 26 °C, 28 °C and 30 °C was significantly higher than that at 24 °C (4.30%, 5.30% and 5.30%). High night temperature of 28 °C affected the grain yield and quality of ZY 309, whereas JH 5 was only affected at 30 °C.

**Table 1:** Grain starch, protein, lipid, and coarse fiber contents of ZY 309 and JH 5 under different night temperature treatments.

Hybrids	Treatment	Grain starch (%)	Grain protein (%)	Grain lipid (%)	Grain coarse fiber (%)
ZY 309	24 °C	71.48 ± 0.35 a	9.60 ± 0.41 c	4.22 ± 0.12 a	3.03 ± 0.05 c
	26 °C	70.20 ± 0.30 a	10.26 ± 0.31 b	4.22 ± 0.08 a	3.18 ± 0.04 b
	28 °C	70.27 ± 0.40 a	10.72 ± 0.60 a	4.08 ± 0.06 b	3.37 ± 0.07 a
	30 °C	69.86 ± 0.40 b	10.70 ± 0.48 a	3.74 ± 0.04 c	3.35 ± 0.06 a
JH 5	24 °C	71.83 ± 0.60 a	8.87 ± 0.32 c	4.24 ± 0.10 a	3.02 ± 0.03 b
	26 °C	70.82 ± 0.30 a	9.08 ± 0.45 b	4.10 ± 0.12 a	3.15 ± 0.05 a
	28 °C	71.58 ± 0.70 a	9.36 ± 0.51 a	3.90 ± 0.10 ab	3.18 ± 0.06 a
	30 °C	69.89 ± 0.25 b	9.75 ± 0.26 a	3.79 ± 0.05 b	3.18 ± 0.05 a

Data are presented as means ± standard deviation (SD) of six biological replicates. Different lowercase letters indicate significant differences among treatments within the same hybrid at *P* < 0.05.

### 3.2 Assimilate allocation and consumption

**Figure 4:**
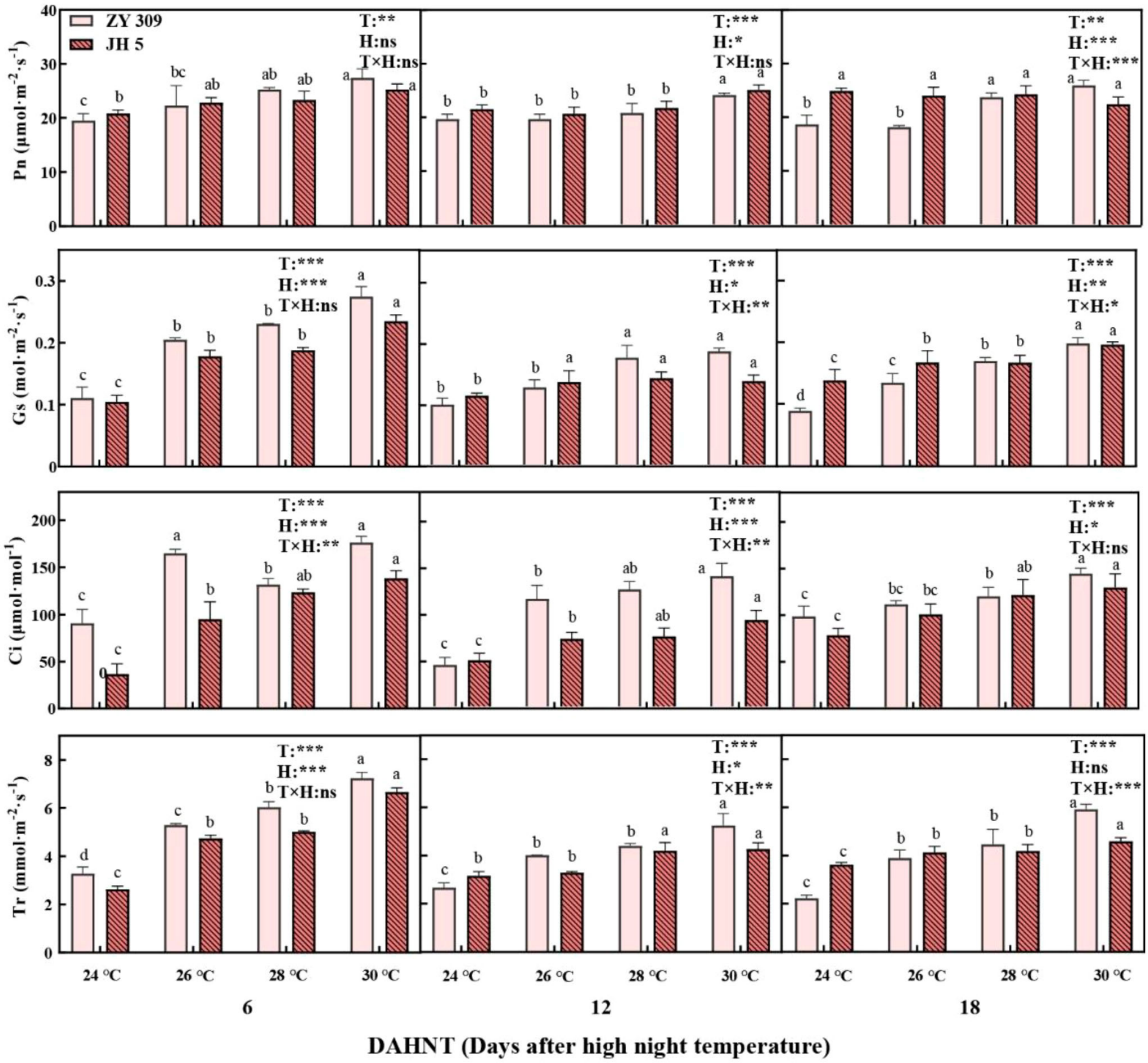
Photosynthetic parameters of ZY 309 and JH 5 under different night temperature treatments. Data are presented as means ± standard deviation (SD) of six replicates at 6, 12, and 18 DAHNT. Different lowercase letters indicate significant differences among treatments within the same hybrid at *P* < 0.05. T represents the temperature factor, H represents the hybrid factor, and T × H indicates the interaction effect. ***, **, and * indicate significance at *P* < 0.001, *P* < 0.01, and *P* < 0.05, respectively; ns indicates no significant difference.

At 6 DAHNT, photosynthesis-related parameters of both hybrids were affected by night temperature treatment (**Figure 4**). At 28 °C and 30 °C, leaf Pn of ZY 309 was significantly increased by 29.49% and 40.51%, respectively. Leaf Pn of JH 5 was significantly increased by 21.10% at 30 °C compared with 24 °C. The 26 °C, 28 °C, and 30 °C treatments significantly increased leaf Gs, Ci, and Tr values in both ZY 309 and JH 5. At 12 DAHNT, Pn of JH 5 and ZY 309 under 30 °C increased by 22.75% and 16.59%, respectively. The 26 °C, 28 °C, and 30 °C treatments significantly increased leaf Gs, Ci, and Tr values in both hybrids, although the magnitude of the response differed between ZY 309 and JH 5. At 18 DAHNT, Pn of ZY 309 was still significantly increased by 27.19% and 39.00% compared with the control at night temperatures of 28 °C and 30 °C, respectively, while there was no significant change in Pn among treatments of JH 5 at night temperature. Both ZY 309 and JH 5 maintained higher Gs, Ci, and Tr after 18 days of night warming under high night temperature treatments.

**Figure 5:**
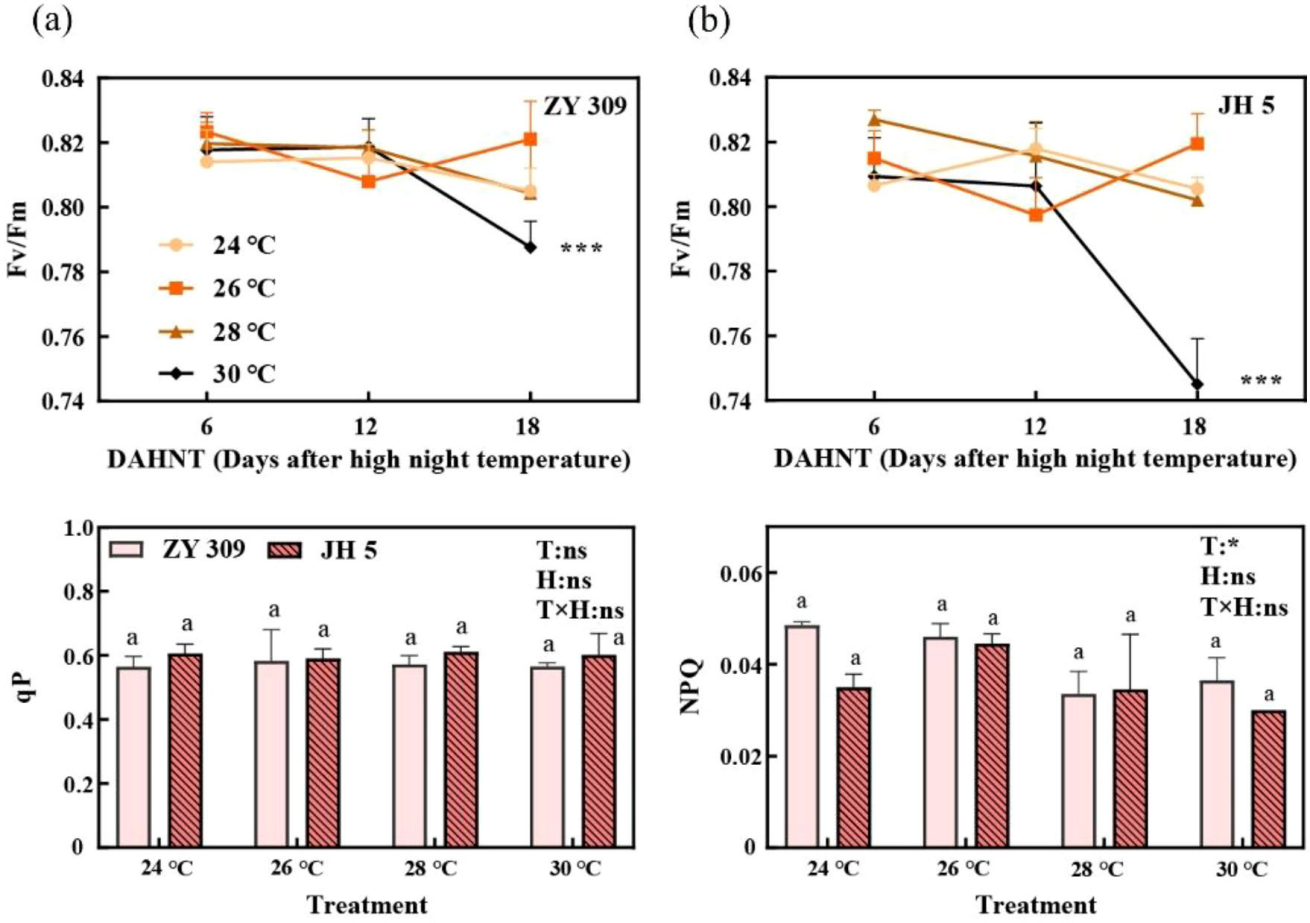
Chlorophyll fluorescence responses of ZY 309 and JH 5 under different night temperature treatments. **(a)** Fv/Fm of ZY 309 and JH 5 under different night temperature treatments. Data are presented as means ± standard deviation (SD) of six replicates at 6, 12, and 18 DAHNT. **(b)** qP and NPQ values of ZY 309 and JH 5 under different night treatments. Data are presented as means ± standard deviation (SD) of six replicates at 18 DAHNT. Different lowercase letters indicate significant differences among treatments within the same hybrid at *P* < 0.05. T represents the temperature factor, H represents the hybrid factor, and T × H indicates the interaction effect. *** and * indicate significance at P < 0.001 and P < 0.05, respectively; ns indicates no significant difference.

Chlorophyll fluorescence parameters showed limited responses to night temperature treatments (**Figure 5**). No significant differences in Fv/Fm were observed among treatments at 6 and 12 DAHNT in either hybrid. At 18 DAHNT, however, Fv/Fm declined significantly under 30 °C, by 2.15% in ZY 309 and 7.51% in JH 5, respectively, compared with the control. In addition, qP and NPQ values at 18 DAHNT were further analyzed and did not differ significantly among treatments in either hybrid.

**Figure 6:**
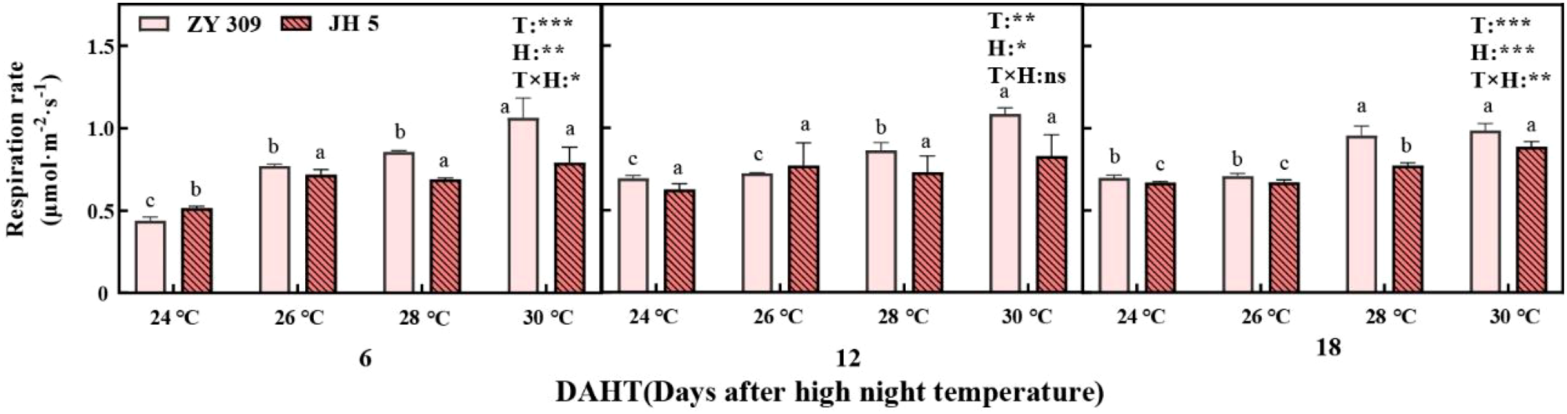
Night respiration rate of ZY 309 and JH 5 under different night temperature treatments. Data are presented as means ± standard deviation (SD) of six replicates at 6, 12, and 18 DAHNT. Different lowercase letters indicate significant differences among treatments within the same hybrid at *P* < 0.05. T represents the temperature factor, H represents the hybrid factor, and T × H indicates the interaction effect. ***, **, and * indicate significance at *P* < 0.001, *P* < 0.01, and *P* < 0.05, respectively; ns indicates no significant difference.

Night respiration rate increased with increasing night temperature in both hybrids, and the response was generally greater in ZY 309 than in JH 5 (**Figure 6**). At 6 DAHNT, respiration rate in ZY 309 increased by 75.43%, 94.88%, and 141.64% under 26 °C, 28 °C, and 30 °C, respectively, whereas the corresponding increases in JH 5 were 39.28%, 33.79%, and 53.54%. At 12 DAHNT, respiration in ZY 309 was significantly increased by 24.35% and 56.34% at 28 °C and 30 °C, while there was no significant difference in the respiratory rate of JH 5. At 18 DAHNT respiration rate of ZY 309 was still significantly increased by 36.81% and 41.49% at 28 °C and 30 °C, and that of JH 5 was significantly increased by 15.62% and 32.60% at 28 °C and 30 °C, respectively.

### 3.3 Physiological response of maize under high night temperature of 30 °C

**Figure 7:**
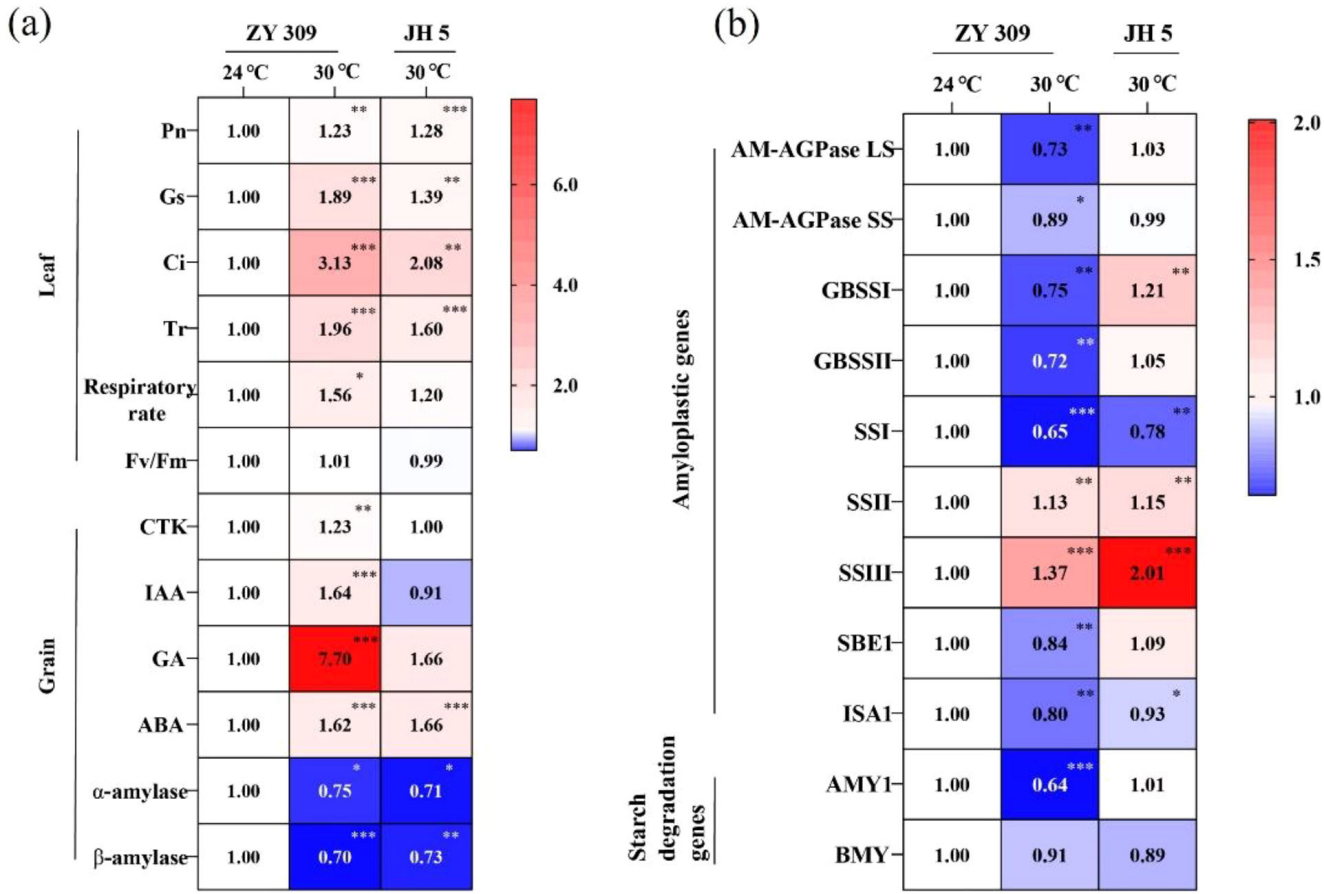
Physiological and molecular responses of ZY 309 and JH 5 under 24 °C and 30 °C night temperature treatments. **(a)** Heat map showing the relative changes in leaf photosynthesis-related parameters, grain hormone contents, and enzyme activities in ZY 309 and JH 5 under 24 °C and 30 °C night temperature treatments. **(b)** Heat map showing the expression of genes involved in starch biosynthesis and degradation in ZY 309 and JH 5 under 24 °C and 30 °C night temperature treatments. In the heat maps, blue and red indicate lower and higher relative values, respectively, as shown in the color scale. ***, **, and * indicate significance at *P* < 0.001, *P* < 0.01, and *P* < 0.05, respectively; ns indicates no significant difference.

At 12 DAHNT, the photosynthetic parameters of ZY 309 were significantly affected by the night temperature of 30 °C compared to JH 5, and the respiration rate of ZY 309 was significantly increased (**Figure 7a**). The night temperature of 30 °C significantly increased CTK, IAA, GA, and ABA hormone levels in ZY 309 grains. ABA in JH 5 grains was also significantly increased, but GA was not affected by night temperature, which was associated with relatively greater heat tolerance in JH 5. The two hybrids significantly decreased the α-amylase and β-amylase activities related to starch degradation. The normalized relative grain hormonal and amylase activity values are provided in supporting information **Supplementary Table 4**.

### 3.4 Expression of genes involved in starch biosynthesis and degradation

Among the amyloplastic genes encoding enzymes involved in starch metabolism, the expression levels of *AM-AGPase LS* and *AM-AGPase SS* were significantly reduced in ZY 309 under the 30 °C night temperature treatment, whereas no significant change was observed in JH 5 (**Figure 7b**). Under higher night temperature, the expression of *GBSSI* and *GBSSII*, which encode granule-bound starch synthase, was also reduced in ZY 309. In contrast, the expression levels of *SSII* and *SSIII* were higher in JH 5 than in ZY 309. The expression levels of *SBE1* and *ISA1* were more strongly affected in ZY 309 than in JH 5 under the 30 °C treatment. Elevated night temperature did not significantly affect the expression of *AMY1* and *BMY* in JH 5, whereas *AMY1* expression was significantly reduced in ZY 309.

## 4 Discussion

### 4.1 Negative effects of high night temperature on maize yield and quality

Previous studies have found that high night temperature during the flowering stage mainly reduces maize yield through its negative effects on kernel number. In particular, Wang et al. (2020) demonstrated that exposure to high night temperature during flowering reduced pollen viability and caused lower kernel number. Kettler et al. (2022) also showed that post-flowering high night temperature impairs maize yield by decreasing kernel number, indicating that disruption of the plant carbon balance is a major driver of yield loss. By contrast, in the present study high night temperature was imposed during the effective grain-filling stage, and the resulting yield reduction was mainly associated with decreased kernel weight rather than kernel number. This suggests that the yield penalty of elevated night temperature depends strongly on the developmental window during which the stress occurs. Notably, the magnitude of this response differed between the two hybrids, with ZY 309 showing a greater reduction in grain yield and kernel weight than JH 5, indicating genotype-dependent sensitivity to elevated night temperature during grain filling.

High night temperature during the effective grain-filling stage reduced maize yield, which is consistent with previous reports showing that daytime heat stress can also reduce maize yield mainly through a decrease in kernel weight (Boehlein et al., 2019; Mayer et al., 2019; Yang et al., 2017). However, these previous studies mainly examined daytime temperatures. Previous studies have suggested that heat stress during grain filling may increase carbon consumption and reduce assimilate accumulation in developing kernels (Impa et al., 2019). In addition, reduced sink activity may further constrain dry matter accumulation during grain filling (Abeledo et al., 2020; Farooq et al., 2011). These possibilities may help explain why yield reduction in the present study was mainly associated with kernel weight rather than kernel number.

According to Wang and Frei (2011), high temperature during the grain-filling stage can reduce starch and lipid content, while increasing protein content. In maize, daytime heat stress during grain development has also been reported to reduce starch content and increase protein content in grains (Gu et al., 2018). In the present study, elevated night temperature also changed starch composition, as indicated by the decline in amylose proportion with increasing night temperature. Previous studies in maize and rice similarly reported that heat stress reduced amylose content and altered starch structure (Lu et al., 1996; Martínez et al., 2019; Umemoto et al., 1999), although contrasting responses have also been observed depending on the environmental conditions and the effects on starch-branching enzymes (Martínez et al., 2017). Notably, the changes in grain quality traits were more pronounced in ZY 309 than in JH 5, suggesting that the sensitive hybrid was less able to maintain normal starch accumulation and composition under elevated night temperature.

### 4.2 High night temperature alters photosynthesis and respiration of maize

Crops gain carbon through photosynthesis during the day and consume it through respiration at night, and the balance between these two processes is critical for growth and grain filling (Azcón-Bieto and Osmond, 1983). Previous studies have shown that the effects of high night temperature on photosynthesis in maize are not uniform across developmental stages and experimental conditions. Wang et al. (2020) reported that high night temperature during flowering did not significantly alter photosynthesis but increased night respiration, whereas Kettler et al. (2022) found that post-flowering night warming increased night respiration and reduced photosynthesis. By contrast, in our study, elevated night temperature was associated with higher photosynthesis in part of the treatments. This discrepancy may reflect differences in treatment intensity, experimental system, and measurement conditions. In particular, Kettler et al. (2022) measured photosynthesis under field conditions at midday, whereas photosynthesis in our study was measured under standardized greenhouse conditions. Therefore, the higher photosynthesis observed here may reflect a daytime photosynthesis-related response at the gas-exchange level rather than the same field-level carbon assimilation response quantified by Kettler et al. (2022). Despite these different daytime photosynthetic responses, these studies support the conclusion that elevated night temperature increases respiratory carbon cost during the reproductive period, thereby disturbing carbon balance.

Daytime high temperature is accompanied by night high temperature, and as night minimum temperature increases, the recovery time of crops to daytime high temperature is reduced, so the “hidden physiological costs” caused by temperature on crops are becoming more and more obvious (Sadok and Jagadish, 2020). High temperatures occurring during the daytime and night affect maize photosynthesis in different ways. A daytime temperature around 30 °C is considered optimal for maize during the middle and late filling period, when leaves maintain a high photosynthetic rate, stable cell membrane integrity and high photosynthetic enzyme activities (Crafts-Brandner and Salvucci, 2002; Sánchez et al., 2014). The suitable temperatures for maize make it possible to accumulate more during the day while avoiding excessive respiratory carbon loss at night. By contrast, elevated night temperature primarily increases respiratory carbon consumption at night, thereby altering the balance between daytime carbon gain and nighttime carbon loss.

An important finding of this study is that the increase in Pn under elevated night temperature did not translate into higher grain yield. In ZY 309, Pn remained 27.19% and 39.00% higher than the control at 28 °C and 30 °C at 18 DAHNT, respectively; however, grain yield decreased by 9.27% and 13.64%, and kernel weight decreased by 6.84% and 10.94% under the same treatments. This apparent discrepancy indicates that instantaneous daytime photosynthetic capacity was not the primary limitation for yield formation under HNT. Instead, the additional carbon fixed during the day may have been offset by increased respiratory carbon consumption at night, leading to a decline in carbon use efficiency for grain filling. In addition, yield formation during the effective grain-filling stage depends not only on source carbon supply but also on the capacity of developing kernels to convert imported assimilates into storage compounds. The downregulation of *AM-AGPase SS* and *AM-AGPase LS* in ZY 309 under 30 °C indicates a weakened capacity for ADP-glucose formation, a key step in starch biosynthesis. Therefore, the yield penalty under HNT likely resulted from the combined effects of increased respiratory carbon cost and restricted sink capacity, rather than from insufficient daytime photosynthetic carbon assimilation alone.

### 4.3 Different starch metabolism in grain affected the response of different hybrids under HNT

High night temperature reduced grain starch proportion in both hybrids, indicating that starch accumulation during grain filling was negatively affected under night warming. However, the transcriptional responses associated with starch metabolism differed markedly between the two hybrids. In ZY 309, several genes involved in starch synthesis, including *AM-AGPase LS*, *AM-AGPase SS*, *GBSSI*, and *GBSSII*, were downregulated under the 30 °C treatment, whereas the corresponding changes were smaller or not significant in JH 5. These results suggest that although starch accumulation was impaired in both hybrids, the disruption of starch synthesis was stronger in the sensitive hybrid ZY 309. ADP-glucose pyrophosphorylase (AGPase) plays a key role in the sequential reactions leading to ADP-glucose formation, a critical precursor for initiating starch synthesis (Impa et al., 2020). Therefore, the reduced expression of *AM-AGPase LS* and *AM-AGPase SS* suggests a lower transcriptional support for starch biosynthesis in ZY 309. Similar effects of heat stress on maize grain metabolism have been reported previously. Wilhelm et al. (1999) showed that combined high day and night temperature during grain filling altered kernel growth and carbohydrate metabolism in maize. Although their treatment involved simultaneous daytime and nighttime heat stress and therefore differs from the night-warming conditions used in the present study, both studies support the view that heat stress during grain filling disrupts starch metabolism and limits assimilate deposition in developing kernels. This interpretation is consistent with the greater reduction in kernel weight observed in ZY 309 under elevated night temperature.

**Figure 8:**
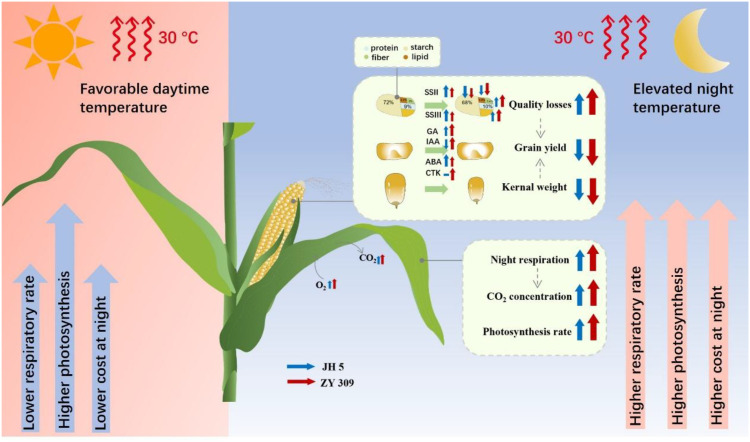
Conceptual framework illustrating the contrasting effects of favorable daytime temperature and elevated night temperature, and the overall response of maize to elevated night temperature during the effective grain-filling stage.

In addition to starch metabolism-related genes, hormone responses also differed between the two hybrids. Under HNT, ZY 309 showed higher CTK, IAA, and GA contents than JH 5, whereas ABA increased in both hybrids. These results suggest that elevated night temperature altered not only grain composition but also the regulatory processes associated with starch accumulation. Taken together, the stronger disturbance of starch-metabolism-related gene expression and hormone regulation in ZY 309 was consistent with its greater reduction in kernel weight and more pronounced changes in grain quality under elevated night temperature. When considered together with the differences in photosynthesis–respiration responses discussed above, these findings suggest that hybrid-specific regulation of carbon use, partitioning, and starch metabolism may contribute to the contrasting tolerance of ZY 309 and JH 5 under HNT. Overall, our results indicate that elevated night temperature impaired starch accumulation and altered its metabolic regulation in both hybrids, with a stronger disturbance in the sensitive hybrid ZY 309 (**Figure 8**).

### 4.4 Limitations and agronomic implications

While the greenhouse system allowed us to isolate HNT responses under controlled conditions, pot confinement may restrict root growth and alter soil water dynamics compared with field conditions (Passioura, 2006). In addition, each night temperature treatment was imposed in a separate greenhouse compartment, so a potential compartment effect could not be completely excluded. Molecular and hormonal analyses were also restricted to the 24 °C and 30 °C treatments at 12 DAHNT. Field studies with replicated warming systems and more sampling are needed to validate these findings.

Despite these limitations, our results suggest that sustained night temperatures of approximately 28–30 °C during effective grain filling may represent a risk range for kernel weight loss, with ZY 309 showing significant yield reduction at 28 °C and JH 5 mainly at 30 °C. This threshold information may help identify high risk periods during grain filling and support the selection of hybrids with lower respiratory carbon loss, more stable starch metabolism, and better kernel weight maintenance under elevated night temperature.

## 5 Conclusion

High night temperature during the effective grain-filling stage reduced maize yield mainly by decreasing kernel weight, with yield loss occurring at 28 °C in ZY 309 and mainly at 30 °C in JH 5. Elevated night temperature also shifted grain composition toward lower starch and lipid contents but higher protein and coarse fiber contents, indicating that grain quality formation was affected together with yield formation. Although daytime photosynthesis increased under elevated night temperature, this increase did not translate into higher yield, suggesting that yield loss was not caused by insufficient photosynthetic capacity alone. Instead, increased night respiration, together with reduced expression of *AM-AGPase SS* and *AM-AGPase LS*, indicates that elevated night temperature may reduce carbon use efficiency and restrict starch biosynthesis in developing kernels. Compared with ZY 309, JH 5 showed greater tolerance to elevated night temperature, which was associated with lower leaf respiration rates and greater stability of starch metabolism. Overall, these results highlight the potential physiological sensitivity of maize to elevated night temperature during the effective grain-filling stage. Further field studies are required to determine whether these physiological responses translate into yield penalties and management implications under agricultural production systems.

## Data Availability

The raw data supporting the conclusions of this article will be made available by the authors, without undue reservation.
