# Supplementary material for: High night temperature during the effective grain-filling stage affects maize yield and quality
Source: Front Plant Sci. 2026 Jul 3;17:1826388. doi: 10.3389/fpls.2026.1826388 (PMC13375488; doi:10.3389/fpls.2026.1826388)
Supplement: Supplementary file 1 [file DataSheet1.pdf]

# Supplementary Materials

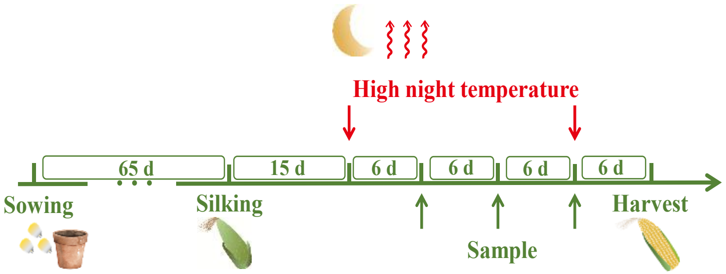


Fig. S1. Growing process and sampling period of potted maize

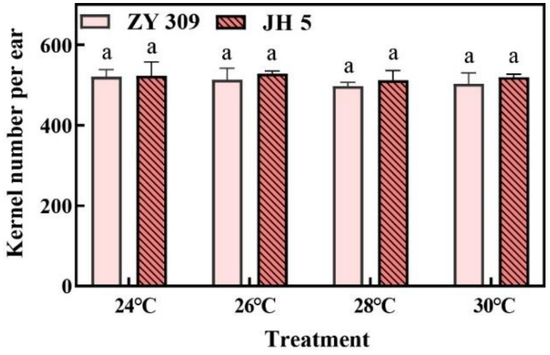


Fig. S2. Kernel number per ear of ZY 309 and JH 5 under different night temperature treatments. Data are presented as means ± standard deviation (SD) of six biological replicates. Different lowercase letters indicate significant differences among treatments within the same hybrid at $P < 0.05$.

Table S1. Coefficient of variation (CV) for yield-related traits of ZY 309 and JH 5 under different night temperature treatments.

| Hybrid | Treatment | Grain yield CV (%) | Kernel weight CV (%) | Kernel number CV (%) |
|---|---|---|---|---|
| ZY 309 | 24 °C | 1.17 | 0.88 | 3.38 |
| | 26 °C | 1.51 | 3.15 | 5.56 |
| | 28 °C | 1.45 | 1.32 | 1.99 |
| | 30 °C | 1.44 | 2.28 | 5.34 |
| JH 5 | 24 °C | 3.63 | 6.10 | 6.62 |
| | 26 °C | 2.45 | 7.43 | 1.14 |
| | 28 °C | 3.82 | 2.75 | 3.87 |
| | 30 °C | 1.06 | 11.05 | 1.50 |

Table S2. List of primers for the genes encoding starch biosynthesis and degradation enzymes used in the qPCR gene expression study.

| **Gene** | **Forward primer (5'-3')** | **Reverse primer (5'-3')** |
|---|---|---|
| *AM-AGPase SS* | GCAAGATACACCATTCAGTAGTTGGAC | GACTGTTCCACTAGGGAGTAAAGCATC |
| *AM-AGPase LS* | GATGAGATCTCGAGGCTGATGTCC | TACACGACGGTGCCGTCCTTGAT |
| *GBSSI* | CTCGCCGCCAACTACGACGTC | TGCTCGGGAACTTCTCCTCCAC |
| *GBSSII* | GTTCCAAGTAGGTTCGAGCCATGT | AGAGCCTCCTCCCACTTCTTTGC |
| *SSI* | GTTTCCCACAGAATAACTGCAGGTTGC | GTATGGTCTTTCGTCATGCCTCGC |
| *SSII* | GAATCAAGTTGGCGAGGATGTCGAC | CAATCTCTTTGCCCTCTGAAACTGCAT |
| *SSIII* | GACTTCTCAGGAAATGTCTCTAGCAG | ACCAGTGCACTTATTCTGACACGGTAC |
| *SBE1* | CGCTCGACGACAAGTTTTCCTTCC | CAAGTGCGGGGTGGAGACAGG |

| | | |
|---|---|---|
| *ISA1* | GTCTTCAACCATACAGCTGAGGGT | GAACCTCTGGTCATTATGGATGCAAG |
| *AMY1* | TTCAACTGGGAGTCGTGGAAG | GCATGTAACCTTCGTTGGAGAC |
| *BMY* | CTAGCCAACTATGTCCAAGTCTACGT | ACTGTGGGATGGGGATGTTGACGA |
| Actin | TGACCGTATGAGCAAGGAG | CCAGACAACTCGCAACTTAG |

*AM-AGPase SS* and *AM-AGPase LS*, genes encoding the small and large subunits of amyloplastic ADP-glucose pyrophosphorylase; *GBSSI* and *GBSSII*, genes encoding granule-bound starch synthase I and II; *SSI*, *SSII*, and *SSIII*, genes encoding starch synthase I, II, and III; *SBE1*, gene encoding starch-branching enzyme 1; *ISA1*, gene encoding isoamylase 1; *AMY1*, gene encoding α-amylase 1; *BMY*, gene encoding β-amylase.

Table S3. The normalized relative gene expression values (in terms of relative fold change to its expression in ZY 309 at 24 °C) for grain-starch biosynthesis and degradation enzymes in grains exposed to 24 °C and 30 °C night temperature treatments at 12 DAHNT. Standard deviation (SD) was calculated from 3 biological replicates.

| **Gene** | **Genotype** | **Relative expression values** | |
|---|---|---|---|
| | | **24 °C** | **30 °C** |
| ***AM-AGPase SS*** | **ZY 309** | 1.00 ± 0.00 | 0.89 ± 0.04 |
| | **JH 5** | 1.15 ± 0.88 | 0.99 ± 0.28 |
| ***AM-AGPase LS*** | **ZY 309** | 1.00 ± 0.00 | 0.73 ± 0.07 |
| | **JH 5** | 1.09 ± 0.09 | 1.03 ± 0.16 |
| ***GBSSI*** | **ZY 309** | 1.00 ± 0.00 | 0.75 ± 0.07 |
| | **JH 5** | 1.11 ± 0.10 | 1.21 ± 0.06 |
| ***GBSSII*** | **ZY 309** | 1.00 ± 0.00 | 0.72 ± 0.08 |

| | | | |
|---|---|---|---|
| | **JH 5** | 1.11 ± 0.03 | 1.05 ± 0.09 |
| ***SSI*** | **ZY 309** | 1.00 ± 0.00 | 0.65 ± 0.10 |
| | **JH 5** | 1.14 ± 0.11 | 0.78 ± 0.33 |
| ***SSII*** | **ZY 309** | 1.00 ± 0.00 | 1.13 ± 0.19 |
| | **JH 5** | 1.25 ± 0.10 | 1.15 ± 0.02 |
| ***SSIII*** | **ZY 309** | 1.00 ± 0.00 | 1.37 ± 0.73 |
| | **JH 5** | 1.19 ± 0.18 | 2.01 ± 0.19 |
| ***SBE1*** | **ZY 309** | 1.00 ± 0.00 | 0.84 ± 0.10 |
| | **JH 5** | 1.11 ± 0.12 | 1.09 ± 0.13 |
| ***ISA1*** | **ZY 309** | 1.00 ± 0.00 | 0.80 ± 0.19 |
| | **JH 5** | 1.23 ± 0.03 | 0.93 ± 0.02 |
| ***AMY1*** | **ZY 309** | 1.00 ± 0.00 | 0.64 ± 0.17 |
| | **JH 5** | 1.14 ± 0.09 | 1.01 ± 0.16 |
| ***BMY*** | **ZY 309** | 1.00 ± 0.00 | 0.91 ± 0.19 |
| | **JH 5** | 1.20 ± 0.10 | 0.89 ± 0.09 |

Table S4. Normalized relative values of grain hormone contents and amylase activities in grains exposed to 24 °C and 30 °C night temperature treatments at 12 DAHNT. Values are expressed as fold changes relative to ZY 309 at 24 °C. Standard deviation (SD) was calculated from three biological replicates.

| **Index** | **Genotype** | **Relative hormonal content and amylase activity** | |
|---|---|---|---|
| | | **24 °C** | **30 °C** |
| **CTK** | **ZY 309** | 1.00 ± 0.00 | 1.23 ± 0.24 |

| | | | |
|---|---|---|---|
| | **JH 5** | 1.20 ± 0.16 | 1.00 ± 0.23 |
| **IAA** | **ZY 309** | 1.00 ± 0.00 | 1.64 ± 1.84 |
| | **JH 5** | 0.73 ± 0.93 | 0.91 ± 1.33 |
| **GA** | **ZY 309** | 1.00 ± 0.00 | 7.70 ± 0.38 |
| | **JH 5** | 2.65 ± 0.17 | 1.66 ± 0.11 |
| **ABA** | **ZY 309** | 1.00 ± 0.00 | 1.62 ± 0.64 |
| | **JH 5** | 1.24 ± 2.93 | 1.66 ± 0.65 |
| **α-amylase** | **ZY 309** | 1.00 ± 0.00 | 0.75 ± 0.01 |
| | **JH 5** | 1.00 ± 0.01 | 0.71 ± 0.00 |
| **β-amylase** | **ZY 309** | 1.00 ± 0.00 | 0.70 ± 0.03 |
| | **JH 5** | 1.25 ± 0.05 | 0.73 ± 0.06 |

Table S5. Results of two-way ANOVA for yield-related, photosynthetic, chlorophyll fluorescence, and respiration traits under different night temperature treatments.

| **Trait** | **Sampling time** | **Factor** | **df** | **F-value** | **P-value** |
|---|---|---|---|---|---|
| Grain yield | Maturity | Temperature | 3 | 28.206 | < 0.001 |
| Grain yield | Maturity | Hybrid | 1 | 93.863 | < 0.001 |
| Grain yield | Maturity | T × H | 3 | 0.575 | 0.642 |
| Kernel weight | Maturity | Temperature | 3 | 11.281 | <0.001 |
| Kernel weight | Maturity | Hybrid | 1 | 34.457 | 0.001 |
| Kernel weight | Maturity | T × H | 3 | 3.084 | 0.040 |
| Kernel number | Maturity | Temperature | 3 | 0.618 | 0.616 |
| Kernel number | Maturity | Hybrid | 1 | 1.410 | 0.258 |

| | | | | | |
|---|---|---|---|---|---|
| Kernel number | Maturity | T × H | 3 | 0.120 | 0.947 |
| Pn | 6 DAHNT | Temperature | 3 | 10.95 | 0.002 |
| Pn | 6 DAHNT | Hybrid | 1 | 0.472 | 0.508 |
| Pn | 6 DAHNT | T × H | 3 | 1.224 | 0.351 |
| Gs | 6 DAHNT | Temperature | 3 | 142.289 | < 0.001 |
| Gs | 6 DAHNT | Hybrid | 1 | 27.146 | < 0.001 |
| Gs | 6 DAHNT | T × H | 3 | 2.554 | 0.114 |
| Ci | 6 DAHNT | Temperature | 3 | 63.915 | < 0.001 |
| Ci | 6 DAHNT | Hybrid | 1 | 76.33 | < 0.001 |
| Ci | 6 DAHNT | T × H | 3 | 7.800 | 0.007 |
| Tr | 6 DAHNT | Temperature | 3 | 365.569 | < 0.001 |
| Tr | 6 DAHNT | Hybrid | 1 | 64.454 | < 0.001 |
| Tr | 6 DAHNT | T × H | 3 | 1.598 | 0.251 |
| Pn | 12 DAHNT | Temperature | 3 | 16.481 | < 0.001 |
| Pn | 12 DAHNT | Hybrid | 1 | 5.891 | 0.029 |
| Pn | 12 DAHNT | T × H | 3 | 0.236 | 0.870 |
| Gs | 12 DAHNT | Temperature | 3 | 25.835 | < 0.001 |
| Gs | 12 DAHNT | Hybrid | 1 | 7.099 | 0.021 |
| Gs | 12 DAHNT | T × H | 3 | 8.479 | 0.003 |
| Ci | 12 DAHNT | Temperature | 3 | 48.675 | < 0.001 |
| Ci | 12 DAHNT | Hybrid | 1 | 59.646 | < 0.001 |
| Ci | 12 DAHNT | T × H | 3 | 9.360 | 0.001 |
| Tr | 12 DAHNT | Temperature | 3 | 42.304 | < 0.001 |
| Tr | 12 DAHNT | Hybrid | 1 | 7.399 | 0.020 |
| Tr | 12 DAHNT | T × H | 3 | 6.868 | 0.007 |

| | | | | | |
|---|---|---|---|---|---|
| Pn | 18 DAHNT | Temperature | 3 | 8.034 | 0.002 |
| Pn | 18 DAHNT | Hybrid | 1 | 17.413 | 0.001 |
| Pn | 18 DAHNT | T × H | 3 | 17.797 | < 0.001 |
| Gs | 18 DAHNT | Temperature | 3 | 34.437 | < 0.001 |
| Gs | 18 DAHNT | Hybrid | 1 | 10.747 | 0.007 |
| Gs | 18 DAHNT | T × H | 3 | 4.769 | 0.021 |
| Ci | 18 DAHNT | Temperature | 3 | 20.463 | < 0.001 |
| Ci | 18 DAHNT | Hybrid | 1 | 5.530 | 0.033 |
| Ci | 18 DAHNT | T × H | 3 | 0.995 | 0.422 |
| Tr | 18 DAHNT | Temperature | 3 | 44.367 | < 0.001 |
| Tr | 18 DAHNT | Hybrid | 1 | 0.002 | 0.964 |
| Tr | 18 DAHNT | T × H | 3 | 15.276 | < 0.001 |
| qP | 18 DAHNT | Temperature | 3 | 0.025 | 0.994 |
| qP | 18 DAHNT | Hybrid | 1 | 2.586 | 0.127 |
| qP | 18 DAHNT | T × H | 3 | 0.161 | 0.921 |
| NPQ | 18 DAHNT | Temperature | 3 | 3.918 | 0.028 |
| NPQ | 18 DAHNT | Hybrid | 1 | 5.168 | 0.083 |
| NPQ | 18 DAHNT | T × H | 3 | 1.525 | 0.281 |
| Respiration rate | 6 DAHNT | Temperature | 3 | 38.244 | < 0.001 |
| Respiration rate | 6 DAHNT | Hybrid | 1 | 11.981 | 0.006 |
| Respiration rate | 6 DAHNT | T × H | 3 | 6.190 | 0.012 |
| Respiration rate | 12 DAHNT | Temperature | 3 | 10.019 | 0.004 |
| Respiration rate | 12 DAHNT | Hybrid | 1 | 6.756 | 0.032 |
| | 12 DAHNT | T × H | 3 | 2.550 | 0.129 |
| Respiration rate | 18 DAHNT | Temperature | 3 | 95.476 | < 0.001 |

| | | | | | |
|---|---|---|---|---|---|
| Respiration rate | 18 DAHNT | Hybrid | 1 | 44.867 | < 0.001 |
| Respiration rate | 18 DAHNT | T × H | 3 | 7.375 | 0.003 |

**Note:** df, degrees of freedom; T × H, interaction between night temperature treatment and hybrid. P-values are shown for the effects of night temperature treatment, hybrid, and their interaction. *, **, and *** indicate significance at $P < 0.05$, $P < 0.01$, and $P < 0.001$, respectively; ns indicates no significant difference.
